# *Pterocarpus santalinus* Selectively Inhibits a Subset of Pro-Inflammatory Genes in Interleukin-1 Stimulated Endothelial Cells

**DOI:** 10.3389/fphar.2021.802153

**Published:** 2022-01-18

**Authors:** Priscilla Natalia, Julia Zwirchmayr, Ieva Rudžionytė, Alexandra Pulsinger, Johannes M. Breuss, Pavel Uhrin, Judith M. Rollinger, Rainer de Martin

**Affiliations:** ^1^ Department of Vascular Biology and Thrombosis Research, Medical University of Vienna, Vienna, Austria; ^2^ Department of Pharmaceutical Sciences, Division of Pharmacognosy, Faculty of Life Sciences, University of Vienna, Vienna, Austria

**Keywords:** *Pterocarpus santalinus*, red sandal wood, inflammation, endothelial cells, E-selectin, TRAF1, NF-kappa B, CX3CL1

## Abstract

Based on the traditional use and scientific reports on the anti-inflammatory potential of red sandalwood, i.e., the heartwood of *Pterocarpus santalinus* L., we investigated its activity in a model of IL-1 stimulated endothelial cells. Endothelial cells were stimulated with IL-1 with or without prior incubation with a defined sandalwoodextract (PS), and analyzed for the expression of selected pro-inflammatory genes. The activity of NF-κB, a transcription factor of central importance for inflammatory gene expression was assessed by reporter gene analysis, Western blotting of IκBα, and nuclear translocation studies. In addition, microarray studies were performed followed by verification of selected genes by qPCR and supplemented by bioinformatics analysis. Our results show that PS is able to suppress the induction of E-selectin and VCAM-1, molecules that mediate key steps in the adhesion of leukocytes to the endothelium. It also suppressed the activity of an NF-κB reporter, IκBα phosphorylation and degradation, and the nuclear translocation of NF-κB RelA. In contrast, it stimulated JNK phosphorylation indicating the activation of the JNK signaling pathway. Gene expression profiling revealed that PS inhibits only a specific subset of IL-1 induced genes, while others remain unaffected. Most strongly suppressed genes were the signal transducer TRAF1 and the chemokine CX3CL1, whereas IL-8 was an example of a non-affected gene. Notably, PS also stimulated the expression of certain genes, including ones with negative regulatory function, e.g., members of the NR4A family, the mRNA destabilizing protein TTP as well as the transcription factors ATF3 and BHLHB40. These results provide mechanistic insight into the anti-inflammatory activity of PS, and suggest that it acts through the interplay of negative and positive regulators to achieve a differential inhibition of inflammatory gene expression.

## Introduction

*Pterocarpus santalinus* L. (Fabaceae) grows as a small tree throughout the tropical regions, especially South-East Asia (Arunakumara et al., 2011), and has been used as an ancient Indian as well as traditional Chinese and European remedy (Navada and Vittal, 2014). Medical applications included the treatment of diverse diseases and conditions such as inflammation, diabetes, skin diseases, headache, jaundice, and wound healing; also gastro- and hepatoprotective, anti-microbial, and hypo-lipidemic effects have been reported (Bulle et al., 2016b; Dahat et al., 2021).

Anti-inflammatory properties of *P. santalinus* extracts and some of its isolated metabolites have been demonstrated in several model systems including, e.g., carrageenan-induced paw edema, TNFα production in lipopolysaccharide-stimulated RAW264 cells, concanavalin A stimulated T cell proliferation, and superoxide anion generation in neutrophils (Cho et al., 2001; Kumar, 2011; Yu et al., 2011).

Inflammation is a common feature of many diseases that can affect almost any tissue and organ; prominent examples include the skin, the gastrointestinal tract, the joints, the liver, and the central nervous and cardiovascular systems (Chen et al., 2018). It involves many different cell types and chemical mediators, however, one common denominator is that immune cells need to exit from the blood vessels into the underlying tissue to fulfill their function. Crossing the endothelial monolayer requires endothelial cells (EC) to express, in response to inflammatory stimulation chemokines, cell adhesion molecules, and others to enable and control this process (Mayer et al., 2004). For this reason pro-inflammatory gene expression has proven highly effective as a primary model to monitor pro- and anti-inflammatory activities, which are to a large extent regulated by the transcription factor NF-κB. NF-κB represents a family of five members, RelA/p65, RelB, c-Rel, NFkB1/p50, and NFkB2/p52 that form homo- and heterodimers. In EC, the predominant form is the RelA/p50 heterodimer. It is activated in response to e.g., IL-1, TNF, LPS, advanced glycation end products, or oxidized lipids through cytoplasmic-to-nuclear translocation of the pre-formed protein after release from its inhibitor IκBα. A key step in the NF-κB signaling pathway is the phosphorylation and ubiquitination-dependent degradation of its inhibitor IκBα, thereby enabling the cytoplasmic transcription factor to translocate to the nucleus (De Martin et al., 2000; Mussbacher et al., 2019).

Based on previous reports on anti-inflammatory activities of PS and preliminary results from our lab we aimed to substantiate this activity in our model system of IL-1 stimulated EC and to provide a more detailed insight into its mode of action on the molecular level. We hypothesize that PS, which constitutes a complex mixture of potentially bioactive compounds may exert specific effects on certain parts of the inflammatory response, a feature that could be desirable for clinical applications.

## Materials and Methods

### Preparation and Characterization of the Heartwood Extract

The heartwood of *P. santalinus* was obtained from Kottas Pharma GmbH, Vienna, Austria (Ch.Nr.: P16301836). A voucher specimen (JR-20190315-A1) is deposited at the Department of Pharmaceutical Sciences, Division of Pharmacognosy, University of Vienna, Austria. A large-scale extract was prepared according to the protocol for the generation of lead-like enhanced (LLE) extracts as previously described in Kratz et al. (2016), adapted from Camp et al. (2012). Briefly, 1 kg of the dried, pulverized heartwood was defatted with 2 L n-hexane (VWR International, Radnor, PA, United States; AnalaR NORMAPUR ACS, ≥95%) for 3 days on a nutator. The obtained n-hexane extract was discarded and the remaining defatted material was extracted with 4 L dichloromethane (CH_2_Cl_2_); VWR International; GPR RECTAPUR, ≥99%) for 3 days on a shaker. The CH_2_Cl_2_ extract was filtered, and the filtrate evaporated on a rotary evaporator. The remaining plant material was extracted two more times with CH_2_Cl_2._ The same procedure was repeated three times with methanol (MeOH). CH_2_Cl_2_ and MeOH extracts were combined and dried under vacuum to obtain 124.44 g of the LLE, i.e., PS. The dried extract (PS) was dissolved in DMSO (Carl Roth; Rotipuran ≥99.8%, p.a.) to a final concentration of 10 mg/ml and stored at −20°C until used. Characterization and dereplication of the extract was done by UPLC-ESI-MS (see Supplementary Figure S1 and Supplementary Table S1).

### Cell Culture

Human umbilical vein endothelial cells (HUVEC) were isolated from umbilical cords using collagenase treatment essentially as described (Jaffe et al., 1973; Zhang et al., 1997). The studies were reviewed and approved by Ethics Commission of the Medical University of Vienna. Written informed consent was provided by the participants’ legal next of kin. Cells were seeded in 75 cm^2^ flasks coated with 1% gelatin (Sigma, St. Louis, MO, United States, #04055) and cultured in M199 medium (Lonza, Basel, Switzerland, #12-119F) with 20% heat-inactivated FBS (Sigma, St. Louis, MO, United States, #F6765), penicillin (100 units/ml), streptomycin (100 μg/ml), (Pen-Strep, Lonza, Basel, Switzerland, #DE17-602E), 2 mM L-glutamine (Sigma; #G7513), 5 units/ml heparin, and 25 μg/ml ECGS (Promocell, Heidelberg, Germany, ECGS/Heparin #C-30140). Cells were passaged at a ratio of 1:3 and used until passage 5 for experiments.

### Antibodies and Reagents

Recombinant human IL-1β was from R&D Systems, Minneapolis, MN, United States (#201-LB). The TAK1 inhibitor (5Z)-7-oxozeaenol: (8-(5-chloro-2-(4-methylpiperazin-1-yl) isonicotinamido)-1-(4-fluorophenyl)-4,5-dihydro-1H-benzo [g] indazole-3-carboxamide was from Sigma (#499610) and used at a final conc of 5 μM. The following antibodies were used: NF-κB p65 (Santa Cruz #sc-372), IκBα (Cell Signaling, Frankfurt, Germany, #9241; 1:1000), phospho-IκBα (Cell Signaling, #2859), and β-actin (Santa Cruz, Heidelberg, Germany, #sc-1616). As secondary antibodies, goat anti-mouse HRP (Invitrogen, Carlsbad, CA, United States, #31432), donkey anti-rabbit IgG HRP-linked whole antibody (Sigma, #GENA934), and goat anti-rabbit IgG conjugates with Alexa Fluor 488 (Invitrogen, #A32723) were used.

### Cytotoxicity Assay

HUVEC were incubated with the indicated concentrations of PS for 6 h, and the toxicity assayed using the Resazorin-based *in vitro* Toxicology Assay Kit (Tox8; Merck, Darmstadt, Germany) according to the manufacturer’s recommendations.

### Quantitative Real-Time PCR

**FIGURE 1:**
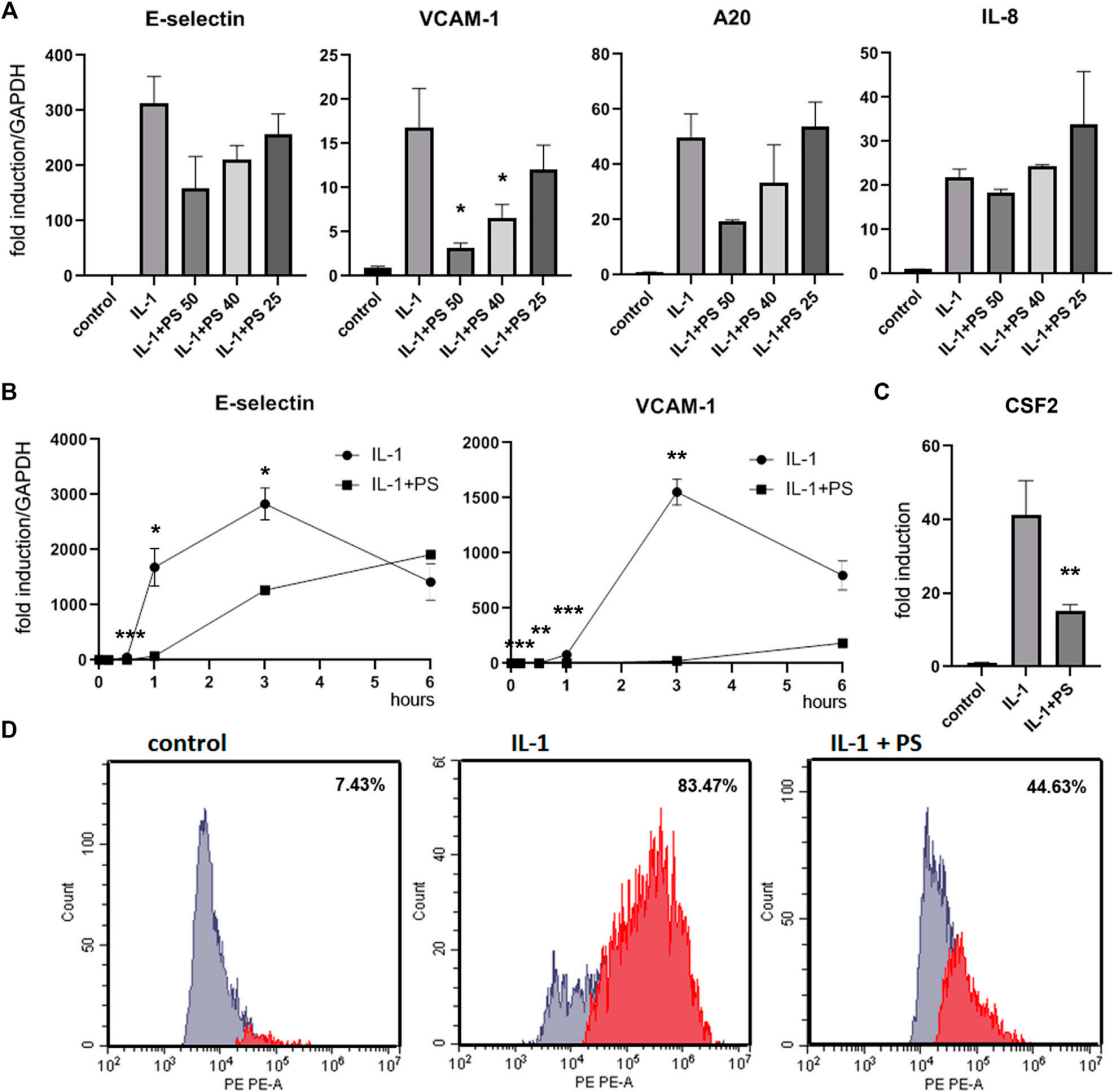
PS suppresses the expression of E-selectin, VCAM-1, A20, and CSF2, but not IL-8. **(A)** HUVEC were pre-incubated for 30 min with 50, 40, and 25 μg/ml PS, then stimulated with 5 ng/ml IL-1 for 2 h and analyzed for E-selectin, VCAM-1, A20, and IL-8 mRNA by qPCR. Relative mRNA levels were normalized to GAPDH and expression levels are depicted as mean fold change +/− SD as compared to unstimulated control cells. **(B)** HUVEC were pretreated with 50 μg/ml PS, then stimulated with IL-1 for the indicated times and analyzed for E-selectin and VCAM-1 as above. **(C)** HUVEC were pre-incubated for 30 min with 50 μg/ml PS, then stimulated with 5 ng/ml IL-1 for 3 h, and supernatants analyzed for CSF2 by ELISA. **(D)** HUVEC were stimulated with 5 ng/ml IL-1 either alone for 3 h or after pre-treatment for 30 min with 50 μg/ml PS, stained with PE-labelled anti-VCAM-1 antibody and analyzed by FACS. Percentages of VCAM-1 positive cells are given in the inserts. *, **, and *** indicate *p* < 0.05, 0.01, and 0.001, respectively, of IL-1 + PS versus IL-1 alone. Note that relative levels of induction may vary between different experiments.

Total RNA was isolated from HUVEC using the PeqGold Total RNA Isolation Kit (VWR International, Radnor, United States, #732-2868) according to the manufacturer’s recommendations. 1 μg RNA was reverse-transcribed using random hexamers (Fisher Scientific, Schwerte, Germany; #SO142) and murine leukemia virus reverse transcriptase (Fisher Scientific, #10338842). Primers were designed using the software “Primer3,” and sequences given in Supplementary Table S2. qPCR was performed using the SsoAdvanced Universal SYBR Green Supermix (BioRad, Vienna, Austria, #1725272) in a StepOnePlus real-time therocycler (Applied Biosystems, Foster City, CA, United States). Relative mRNA expression was normalized to GAPDH. Triplicate samples were analyzed except for Figure 1A where duplicates were performed. Fold changes in mRNA expression were calculated according to the 2-ΔΔCt method (Livak and Schmittgen, 2001).

### FACS Analysis

HUVEC were grown to post-confluency in 6-well plates, pre-treated for 30 min with 50 μg/ml PS or left untreated, then stimulated with 5 ng/ml IL-1β for 3 h. Cells were harvested by trypsinization, stained with anti-VCAM1 antibody (anti-CD106-PE, #305806, Biozym Scientific GmbH, Vienna, Austria) according to the recommendation of the manufacturer, fixed with paraformaldehyde and analyzed on a Cytoflex S (Beckman Coulter, Brea, CA, United States) instrument.

### Western Blotting

HUVEC grown to post-confluency in 6-well plates. Following pre-incubation for 30 min with 50 μg/ml PS, they were stimulated with IL-1β (5 ng/ml) for the periods of time indicated in the Figures. Cells were lysed in Laemmli buffer, and Western analysis for IκBα and p IκBα, as well as β-actin, was performed as described using 10% SDS-PAGE (Seigner et al., 2018). For re-probing, membranes were stripped in 60 mM Tris pH = 7.0/2% SDS/0.7% β-mecaptoethanol for 10 min at 50°C. Densitometric analysis was done with ImageJ.

### Transfection and Reporter Gene Assays

HUVEC in the exponential growth phase were trypsinized and transfected by electroporation using a BioRad Gene Pulser with the settings 200 V/960 μF. 2 × 10^6^ cells were electroporated in 400 μl RPMI medium in 0.4 cm cuvettes with a total of 10 μg plasmid DNA. Plasmids were pNL3.2.NF-κB-RE (Promega, Madison, WI, United States) and pmaxGFP (Amaxa/Origene, Rockville, MD, United States). Cells were seeded into 6-well plates and grown for 2 days before stimulation. Luciferase levels were analyzed using the NanoGlo Luciferase Assay (Promega, #N1110) according to the manufacturer’s instructions and normalized to EGFP fluorescence.

### Enzyme-Linked Immonosorbent Assay

Levels of CSF2 (GM-CSF) were determined by ELISA (ELISA MAX Deluxe Set Human GM-CSF, #B432004, Biozyme Scientific GmBH, Hessisch Oldendorf, Germany) from supernatants of HUVEC pre-treated with 50 μM PS for 30 min and stimulated with 5 ng/ml IL1 for 3 h as indicated in Figure 1C.

### Microarray and Bioinformatic Analysis

Total RNA was isolated using the RNeasy Plus Micro Kit (Qiagen, Hilden, Germany) including DNAse digestion. Analysis was done by the Core Facilities of the Medical University of Vienna. Labelling was performed with the WT Plus Labeling Kit (#902280, ThermoFisher, Waltham, MA, United States), and probes hybridized to Human Gene 2.0 ST Arrays (#902113, ThermoFisher). Data were deposited in the GEO database (accession no. GSE178106), where additional experimental details are described. Experiments were performed in triplicates, and samples pooled for the microarray analysis. The heat map was generated using Genesis software (Institute of Genetics and Bioinformatics, Graz University of Technology). Transcription factor motif analysis was performed using NetworkAnalyst 3.0 (https://www.networkanalyst.ca) with the Encode database. PS responsive genes (Figure 4A and Supplementary Table S3) that were induced by IL-1 > 3-fold and down-regulated by PS > 3-fold were included. The control set consisted of genes with an IL-1 induction > 2-fold and a regulation by PS between 0.9–1.1-fold (Supplementary Table S3).

### Statistical Significance Calculations

Differences between samples were analyzed by ordinary one-way ANOVA using Graph Pad Prism software (San Diego, CA, United States). Dunnett´s multiple comparisons test was added for dose-dependency in Figure 1A. *, **, and *** indicate *p* < 0.05, 0.01, and 0.001, respectively.

## Results

In previous studies, we have analyzed the anti-inflammatory activity of twenty selected herbal extracts using the reduction of the IL-1 stimulated expression of the cell adhesion molecule E-selectin in human umbilical vein endothelial cells (HUVEC) as readout (Lammel et al., 2020). Here, based on the strength and robustness of its inhibitory action, we have chosen PS for further in-depth analysis. The extract was prepared according to for the generation of lead-like enhanced extracts as described in Materials and Methods, and characterized by UPLC and identification of 6 main compounds (Supplementary Figure S1). No toxicity was observed at a concentration of 50 μg/ml (Supplementary Figure S2), so the subsequent experiments were performed at this or lower concentrations. PS tested at 50, 40 and 25 μg/ml decreased the IL-1 induced expression of the mRNAs of E-selectin and another cell adhesion molecule, VCAM-1, as well as the inhibitor of apoptosis A20 in a dose-dependent manner. However, IL-8, a prominent cytokine in the inflammatory process was, apart from a slight decrease at 50 μg/ml PS, not significantly affected (Figure 1A). A kinetic analysis showed that mRNA levels of both cell adhesion molecules were suppressed by the PS extract, with highest effects between 1 and 3 h post stimulation (Figure 1B). In order to determine the effect of PS on the levels of selected proteins we performed an ELISA for a soluble factor, CSF2 (Figure 1C), one of the top PS-regulated genes in the microarray analysis (see below), and a FACS analysis of a membrane-stemming molecule, VCAM-1 (Figure 1D). Both analyses confirmed the suppressive effect. Moreover, during the initial characterization of the extract, E-selectin protein levels have been assayed by cell ELISA and found to be down-regulated by PS (Lammel et al., 2020).

**FIGURE 2:**
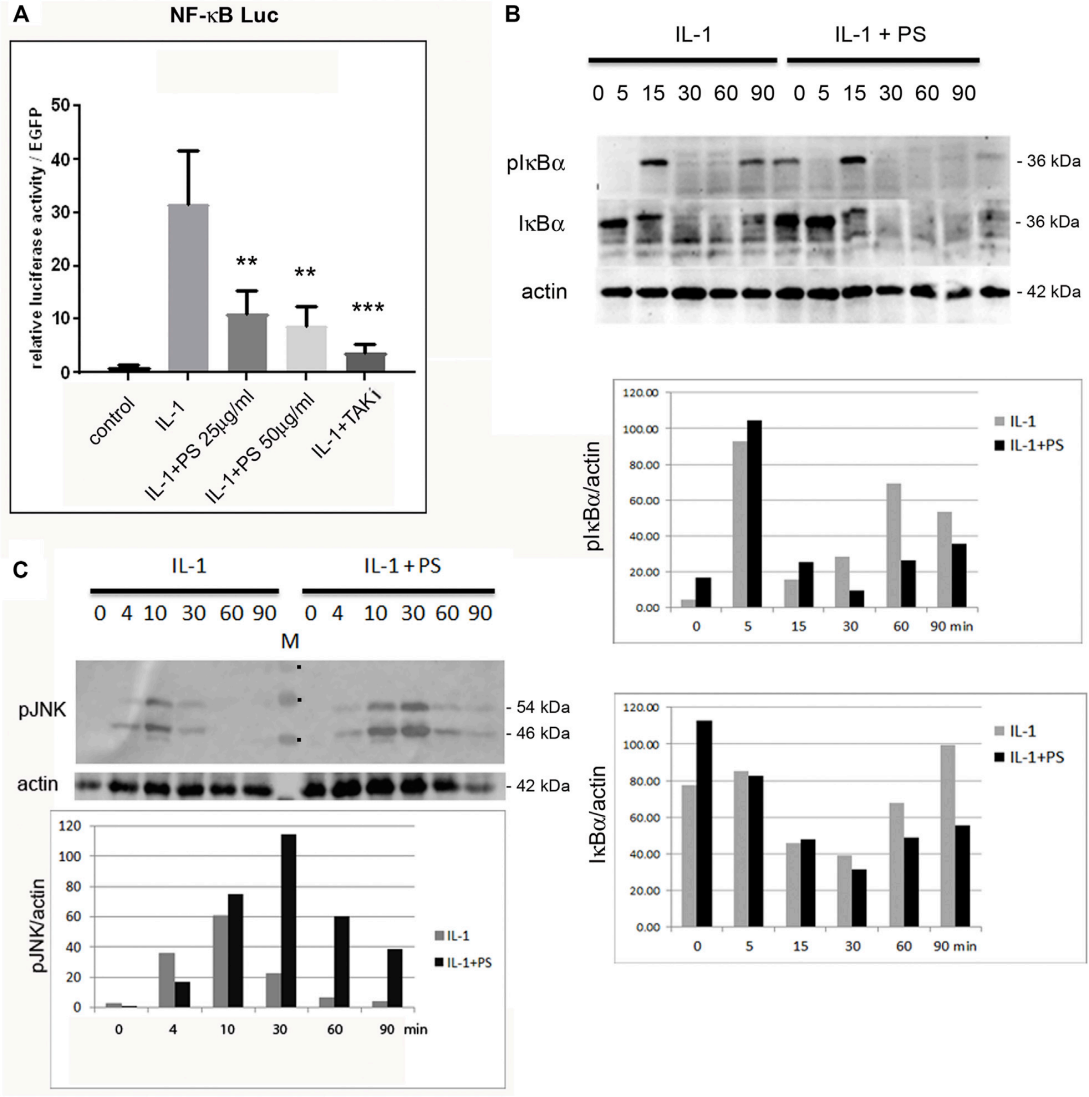
PS inhibits the NF-κB, but enhances the JNK activity. **(A)** HUVEC were transfected with a NF-κB luciferase reporter construct (NF-κB luc) and EGFP as internal control, pretreated for 30 min with 25 or 50 μg/ml PS or 5 μM TAK1 inhibitor (TAKi) as positive control and stimulated with 5 ng/ml IL-1. Control: unstimulated cells. Luciferase levels were determined after 16 h and are shown as relative luciferase levels normalized to EGFP. Triplicate samples were analyzed. **p < 0.01, ***p < 0.001 as compared to IL-1 stimulation; **(B)** HUVEC were either treated with 5 ng/ml IL-1 alone (left) or pre-incubated with 50 μg/ml PS for 30 min following stimulation with IL-1 (right) for the indicated times and analyzed for phospho-IκBα, IκBα, and β-actin by Western blotting. Approximate MWs of the proteins are given on the right. A quantification is given below. **(C)** Western analysis of HUVEC treated with 50 μg/ml PS for 30 min followed by stimulation with 5 ng/ml IL-1 for the indicated times in minutes. Samples were analyzed for phospho-JNK (pJNK) and β-actin. The approximate MW of the two pJNK bands are indicated on the right. M: marker, the three visible bands of 40, 55 and 70 kDA are indicated by dots. Lower panel: quantification of pJNK (average of the p46 and p54 bands) normalized to β-actin.

Since NF-κB is a main regulator of pro-inflammatory genes in EC we assayed its activity using reporter gene analysis. A NF-κB minimal promoter-luciferase reporter was transfected into HUVEC followed by stimulation with combinations of IL-1 and PS. As shown in Figure 2A, PS inhibited the IL-1 induced activity of the reporter gene in a dose-dependent manner, however, not to the same extent as an inhibitor of TAK1, a mitogen-activated protein 3 (MAP3) type kinase in the NF-κB signaling pathway that we used as positive control. Furthermore, we analyzed the kinetics of IκBα phosphorylation and degradation using Western blotting. Following IL-1 stimulation, IκBα was phosphorylated within 5 min, then degraded to undetectable levels, and re-synthesized after 60 min as demonstrated by staining with anti-pIκBα and anti-total IκBα antibodies (Figure 2B, first and second panel, respectively). This is in accordance with previous studies indicating a second wave of activation approximately 1 h after stimulation (Hoffmann et al., 2002; Winsauer and de Martin, 2007). However, in the early phase of IL-1 stimulation, no differences between control- and PS-treated samples were observed. Pre-treatment with PS alone for 30 min even caused a small increase in IκBα levels. This might be due to an initial stress response of the extract [as also supported by the increased levels of pJNK (Figure 2C)]. Given the complex composition of herbal extracts it appears feasible that both positive and negative factors may contribute to a biological effect where, depending on the time of incubation, the one or the other could prevail. In contrast, PS largely prevented the second wave of IκBα re-synthesis and phosphorylation as seen at 60 and 90 min. This indicates that PS acts in part by interfering with the later phase of NF-κB signaling at a point of the pathway at or above the level of IκBα. However, another signaling pathway known to be activated during the inflammatory response, JNK (Kaminska, 2005), was found to be stimulated, as shown by the enhanced and prolonged phosphorylation of JNK (Figure 2C).

**FIGURE 3:**
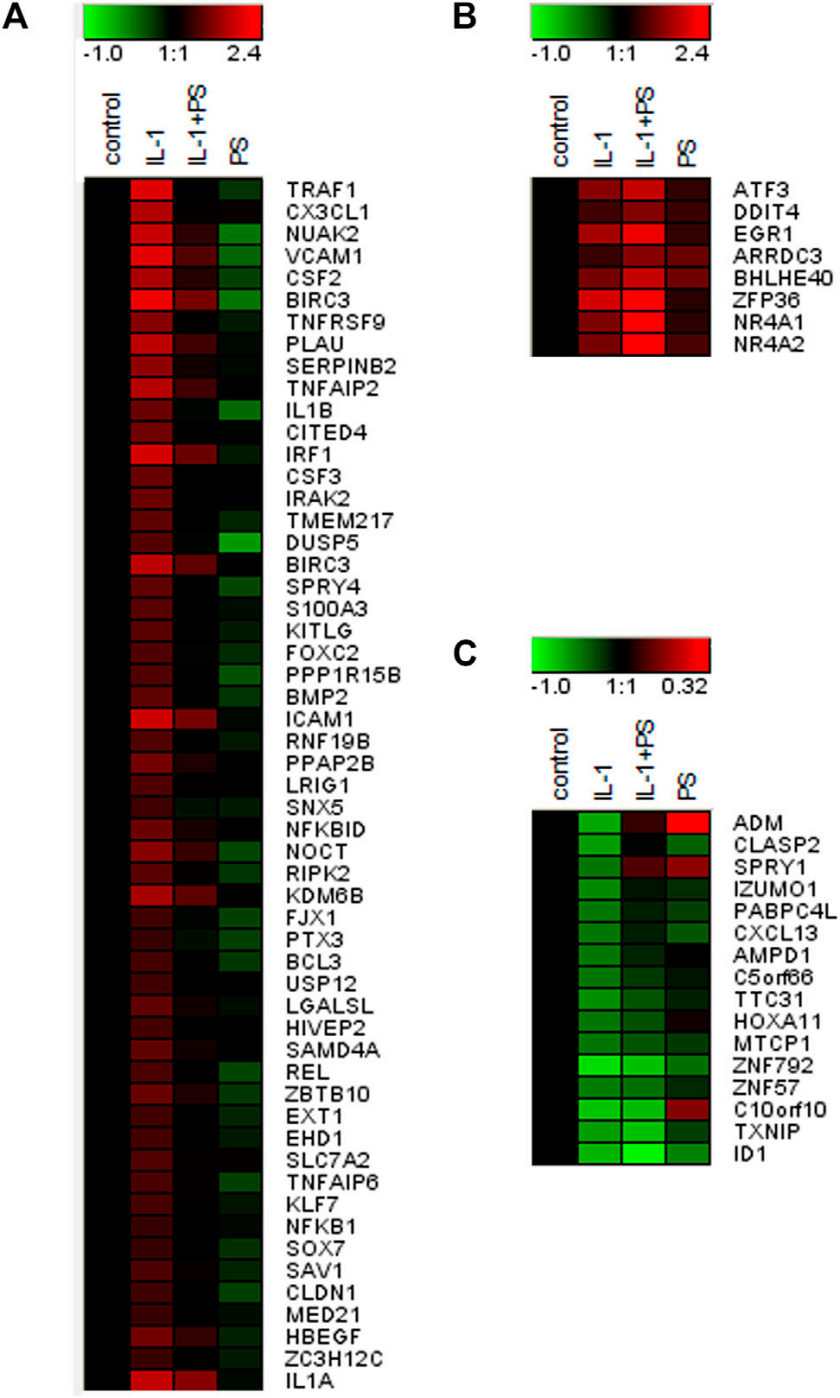
Gene expression profile of PS + IL-1 vs. IL-1 treated HUVEC. A heat map of genes (Official Gene Symbols; gene names are given in Supplementary Table S3) that are regulated more than 3-fold by PS + IL-1 as compared to IL-1 alone is shown. Cells were pre-treated with 50 μg/ml PS for 30 min and then stimulated for 2 h with 5 ng/ml IL-1. **(A)** up-regulation by IL-1, down-regulation by PS; **(B)** up-regulation by IL-1 and further up by PS; **(C)** down-regulation by IL-1, further regulation by PS (up or down). Colors indicate up-regulation (red) and down-regulation (green), logarithmic scale. Note that the scale was adjusted between **(A)**, **(B)**, and **(C)** for optimal visualization.

**FIGURE 4:**
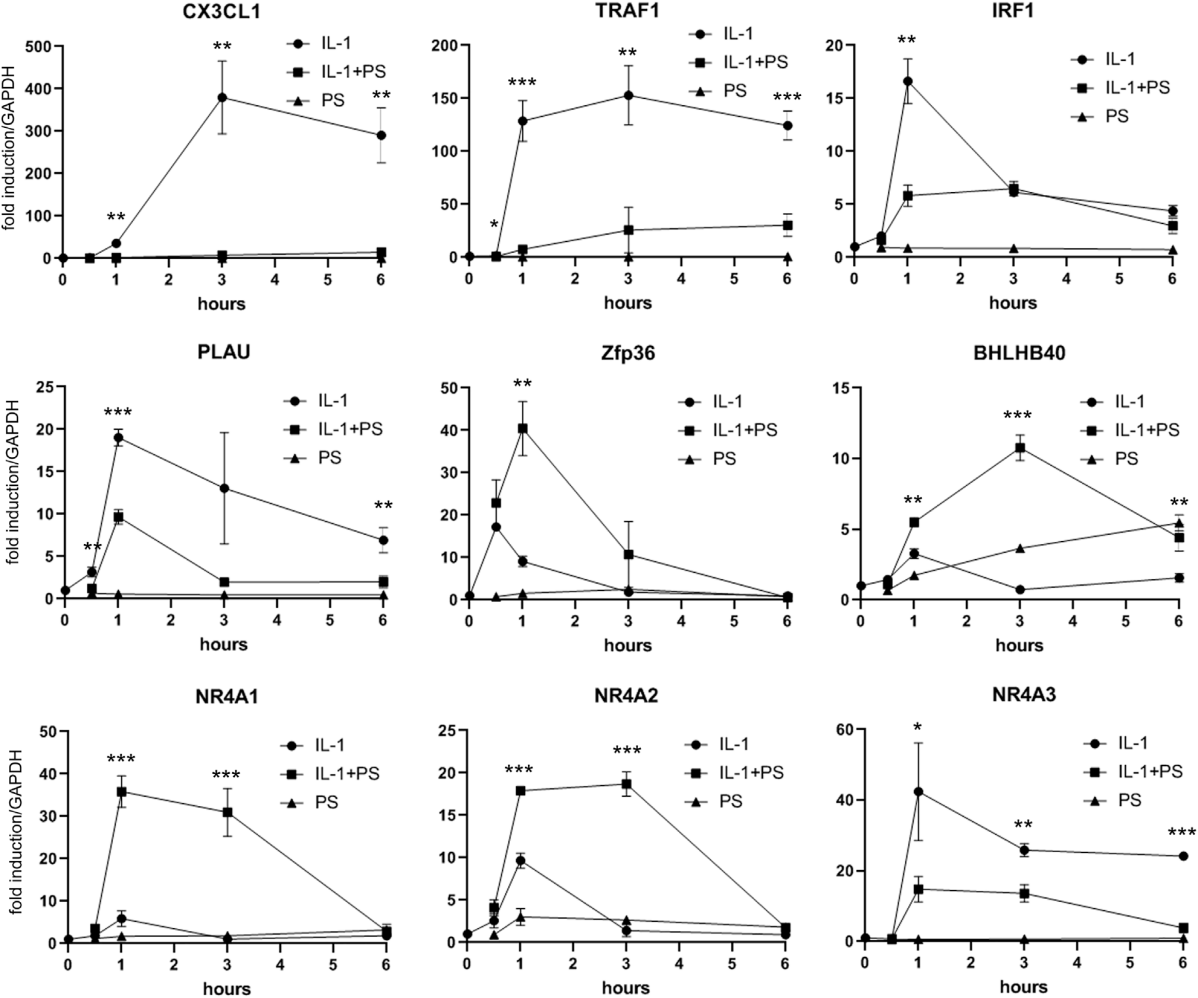
qPCR analysis of selected PS regulated genes. HUVEC were stimulated with 5 ng/ml IL-1 either alone or after pre-treatment for 30 min with 50 μg/ml PS, and analyzed by qPCR as indicated. *, **, and *** indicate *p* < 0.05, 0.01, and 0.001, respectively, of IL-1 + PS versus IL-1 alone.

Encouraged by our initial finding of a differential effect of PS towards pro-inflammatory gene expression (i.e., E-selectin and VCAM-1 versus e.g., IL-8, one of the most prominent and abundant genes in EC), we aimed to obtain a comprehensive overview over the effect(s) of PS. Therefore, expression profiling was performed comparing 1) unstimulated cells (i.e., control), 2) IL-1 treated cells, 3) IL-1 plus PS treated cells and 4) PS only-treated cells. At a threshold of 3-fold stimulation of IL-1 vs. control, 161 genes were up-regulated and 16 down-regulated (lowering the threshold to 2-fold resulted in 362 up- and 201 down-regulated genes), confirming previous studies (Mayer et al., 2004). The >3-fold regulated genes were further sorted by their regulation by PS. Figure 3A shows those genes whose IL-1 response is down-regulated at least 3-fold by PS (a total of 55 genes), with CX3CL1 and TRAF1 showing strongest inhibition; note that in addition, many genes were down-regulated below their basal level by PS alone. In contrast, eight IL-1 stimulated genes were further up-regulated >3-fold by PS (Figure 3B). In panel **C**, 16 genes that were down-regulated by IL-1 (>3-fold) are shown, the extent of further regulation (up or down) by PS is indicated by color. Names for the official gene symbols are given in the Supplementary Table S3. Genes that were either highly regulated or could be of special interest for certain biological aspects, e.g., interferon regulatory factor (IRF1) and plasminogen activator (PLAU) were selected for a more detailed kinetic analysis by qPCR, demonstrating accordance with the results of the microarray experiment (Figure 4).

**FIGURE 5:**
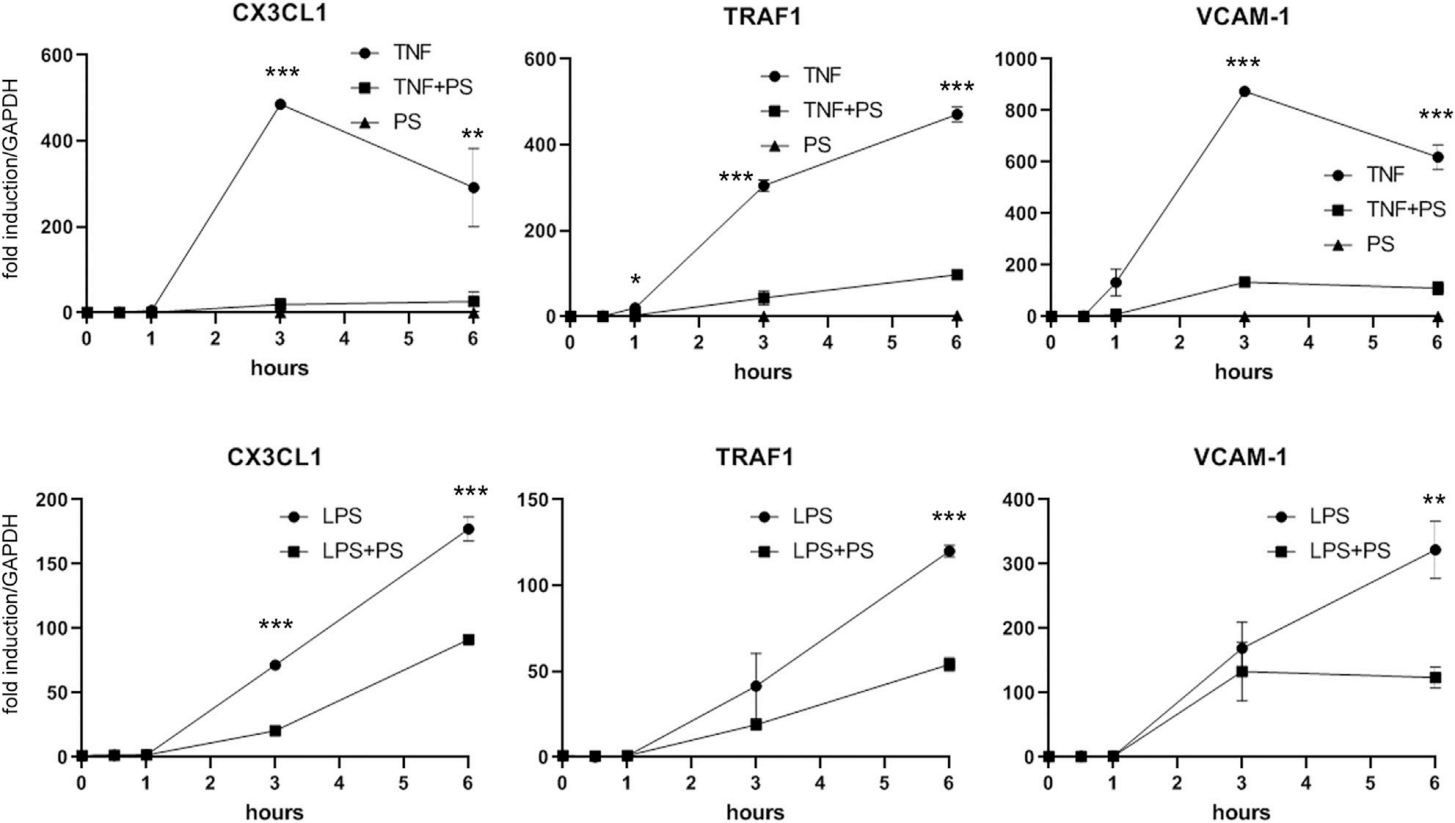
PS suppresses the TNF- or LPS-stimulated expression of CX3CL1, TRAF1, and VCAM-1. HUVEC were stimulated with 2.5 ng/ml TNF (upper panels) or 500 ng/ml LPS (lower panels) either alone or after pre-treatment for 30 min with 50 μg/ml PS and analyzed by qPCR as indicated. *, **, and *** indicate *p* < 0.05, 0.01, and 0.001, respectively, of IL-1 + PS versus IL-1 alone.

Besides IL-1, other pro-inflammatory stimuli including TNF or bacterial lipopolysaccharide (LPS) can activate EC to express a similar set of genes. Although binding to different receptors and using in part different receptor-specific adaptors, the respective signaling pathways that lead to NF-κB converge at the level of the IKK complex (Mercurio et al., 1997). Thus, assuming that PS acts at least in part though NF-κB inhibition, we investigated whether PS would attenuate also TNF- and LPS-stimulated gene expression. As shown in Figure 5, induction of the three genes tested (i.e., CX3CL1, TRAF1, and VCAM-1) was diminished by PS; in the case of LPS, which evokes a slower response as compared to IL-1 and TNF, the inhibition was less pronounced over the observed period.

Given the fact that the IL-1 inducible genes could be grouped into two subsets of PS- responsive and non-responsive ones, we sought to determine the underlying molecular mechanism(s). As a first working hypothesis, we assumed that at least the vast majority of this differential regulation takes place on the transcriptional level, and analyzed the two subsets in regard to the presence or absence of transcription factor binding sites in their promoter regions. We used Motif search of the NetworkAnalyst software package with the Encode database (see *Materials and Methods* section); this tool also offers the identification of target gene-micro RNA interactions. As shown in Table 1, the most prominent binding motif was represented by different variations of the NF-κB site. However, since only a small preference in the subset of PS responsive genes was found (all NF-κB sequences together account for 0.83 vs. 0.63% in the control, when normalized for the different numbers of genes in the two sets), this suggests that NF-κB may only partially account for the observed effects of PS. Two other sites, STAT5A and the target of MIR23 A,B were specifically present in the PS-responsive set. However, only 6 and 7 genes, respectively, contained these sites, suggesting that also these may not be the only ones responsible for the PS effect. Therefore, it can be expected that additional mechanisms affecting different genes are operative. This pluri-mechanistic action is likely to be a result of the interplay from several PS constituents endowed with distinct biological profiles.

**TABLE 1:** Analysis of potential PS-responsive transcription factors and microRNAs.

Pathway PS	Total	Expected	Hits	*p*-Value	FDR
V$NFKAPPAB_01	251	0.838	9	1.19E-07	9.91E-05
V$NFKB_Q6_01	232	0.774	7	1.08E-05	0.00427
V$NFKB_Q6	254	0.848	7	1.94E-05	0.00427
V$CREL_01	256	0.854	7	2.04E-05	0.00427
GGGNNTTTCC_V$NFKB_Q6_01	134	0.447	5	8.05E-05	0.0135
V$NFKAPPAB65_01	237	0.791	6	0.000128	0.0179
V$STAT5A_01	251	0.838	6	0.000176	0.021
AATGTGA MIR-23A, B	419	1.4	7	0.000441	0.0461
TATAAA_V$TATA_01	1300	4.32	12	0.000871	0.0809
V$NFKB_C	263	0.878	5	0.00177	0.129
V$GATA_C	266	0.888	5	0.00186	0.129
V$NFAT_Q4_01	266	0.888	5	0.00186	0.129
V$STAT3_01	22	0.0734	2	0.00241	0.155
**Pathway control**	**Total**	**Expected**	**Hits**	***p*-Value**	**FDR**
V$CREL_01	256	0.436	5	6.11E-05	0.034
ACTTTAT MIR-142-5P	288	0.491	5	0.000107	0.034
GGGAGGRR_V$MAZ_Q6	2270	3.87	12	0.000122	0.034
ACTGCCT MIR-34B	219	0.373	4	0.000473	0.0767
V$NFKAPPAB65_01	237	0.404	4	0.000637	0.0767
V$CHOP_01	238	0.406	4	0.000647	0.0767
V$CREBP1_Q2	254	0.433	4	0.000826	0.0767
V$NFKB_Q6	254	0.433	4	0.000826	0.0767
V$NFKB_C	263	0.448	4	0.00094	0.0767
CAGTATT MIR-200B, C MIR-429	469	0.799	5	0.00101	0.0767
V$MYCMAX_B	268	0.457	4	0.00101	0.0767
RNGTGGGC_UNKNOWN	766	1.31	6	0.00147	0.102
CAGGTA_V$AREB6_01	792	1.35	6	0.00174	0.112

Among the IL-1 responsive genes a subset of PS responsive (pathway PS), and one of PS non-responsive genes (pathway control) were defined as outlined in *Materials and Methods* section and shown in the Supplementary Table S3. Their promoter regions were analyzed for transcription factor binding sites and for target gene-microRNA interactions using NetworkAnalyst 3.0.

Sites that are enriched in the PS responsive gene set are indicated in blue, those that are common in both sets in red. Only the top 13 sites (by significance) are shown.

FDR, false discovery rate.

## Discussion

Despite the long-standing traditional use of *P. santalinus,* only few *in vivo* and *in vitro* studies on its anti-inflammatory activity, the latter using macrophages, neutrophils, and also T cells, have been performed (Cho et al., 2001; Kumar, 2011; Wu et al., 2011). Despite these studies, the underlying molecular mechanism(s) remain poorly understood. From a pre-characterized set of extracts of herbal and fungal origin, *P. santalinus* has caught our attention due to its strong and robust effect in a model of IL-1 stimulated EC (Lammel et al., 2020). EC play a key role in the inflammatory response due to their control of immune cell transmigration, which requires the tightly regulated expression of cell adhesion molecules, interleukins, and chemotactic factors.

Initially, we assayed IL-1 stimulated E-selectin expression, and found a dose-dependent inhibitory effect of PS on the mRNA level. Inhibition of another adhesion molecule, VCAM-1, was even more pronounced, however, IL-8, an important gene in the inflammatory response, was not affected. A more detailed kinetic analysis of the two adhesion molecules confirmed these findings, as well as the inhibition of VCAM-1 on the protein level (Figure 1).

Since NF-κB is a prominent regulator of pro-inflammatory genes in EC and has been demonstrated to be a target of many natural products (Kim et al., 2007; Seigner et al., 2019; Lammel et al., 2020), we reasoned that this transcription factor might also be affected by PS. A reporter gene analysis confirmed this hypothesis (Figure 2A), and was further substantiated by IκBα phosphorylation and degradation (Figure 2B). However, PS appeared to display little effects on the early stages of NF-κB activation, whereas IκBα phosphorylation and degradation were strongly diminished at 60–90 min. This indicates that primarily, the signaling downstream of IκBα degradation was affected by PS, whereas its resynthesis and degradation, which is indicative of a second wave of activation is suppressed. The observation that NF-κB activation occurs in waves has been described by Hoffman and Baltimore in T-cells (Hoffmann et al., 2002) and also by our lab in endothelial cells and fibroblasts (Winsauer et al., 2008; Seigner et al., 2019). Here, preliminary data indicated that nuclear translocation 15 min after stimulation was not affected (data not shown), suggesting that post-translational modifications of NF-κB could be targeted by PS. Several modifications that modulate NF-κB activity have been reported and include phosphorylations, acetylations, or S-nitrosylations (Anrather et al., 2005; Perkins, 2006; Sen et al., 2012). Especially the latter can affect redox-sensitive residues, which could be subject to, e.g., polyphenolic compounds with anti-oxidant properties that are present in PS (Bulle et al., 2016a). Alternatively, DNA binding of the transcription factor could be affected, e.g., by IκBα that has been reported to shuttle between nucleus and cytoplasm and prevent DNA binding (Kerr et al., 1991; Birbach et al., 2002). The precise mechanism should be subject to further investigation, preferentially with isolated compounds. We also assayed for activation of another prominent pathway, JNK that is operative during the inflammatory response (Kaminska, 2005). Surprisingly, activation of this pathway was enhanced and prolonged, as shown by Western analysis of phospho-JNK (Figure 2C).

This finding and the initial observation of differential effects on inflammatory gene expression prompted our subsequent analysis of global gene expression in response to PS. When comparing IL-1 treatment of HUVEC with the combination of IL-1 plus PS, striking differences in the susceptibility of IL-1 induced genes towards PS became apparent (Figure 3). For example, levels of the two most strongly affected genes, TRAF1 and CX3CL1, were reduced by 98 and 95%, respectively, by PS, whereas one of the most prominent pro-inflammatory chemokines in EC, IL-8, remained essentially unchanged, in accordance with Figure 1A. CX3CL1, also known as fractalkine, is an atypical member of the chemokine family. It is a strong chemoattractant for monocytes and T cells, and in its membrane-bound form also for neutrophils (Bazan et al., 1997). TRAF1 is part of the cytoplasmic part of the TNF receptor signaling complex and thus plays a part in TNF signaling by coupling directly to IKK2 (Sughra et al., 2010). As an inducible gene it represents a positive feedback loop for TNF but also LPS signaling (Su et al., 2006; Abdul-Sater et al., 2017); its inhibition by PS would predict to counteract this amplification. However, since also IL-1 and LPS signaling were affected, other mechanisms are likely operative or prevail in this situation. Other induced genes of note include PLAU (urokinase-type plasminogen activator), an important regulator of fibrinolysis, the colony-stimulating factors G-CSF and GM-CSF (CSF2) that serve as differentiation factors for granulocytes and macrophages during the inflammatory episode (Quesenberry and Gimbrone, 1980), BIRC3 (cIAP2), an inhibitor of apoptosis (Guo et al., 2014), and many others. It is noteworthy that at least two genes, TNFAIP2 and A20, the latter also displaying anti-apoptotic properties, have been found to inhibit NF-κB signaling (Cooper et al., 1996; Thair et al., 2016). Of special interest is IRF1 (interferon-regulatory factor 1), a transcription factor for interferon-β (Miyamoto et al., 1988), since it has been demonstrated to cross-talk with NF-κB upon e.g., VCAM-1 expression, (Neish et al., 1995), and DUSP5, a dual-specificity phosphatase that serves to terminate MAP kinase signaling (Kovanen et al., 2003). Inhibition of its expression could explain the prolonged activation of JNK signaling seen upon PS treatment (Figure 2C).

On the other hand, a number of genes were up-regulated by PS, e.g., the nuclear hormone receptors NR4A1-3 (Rodríguez-Calvo et al., 2017), the transcription factors ATF3 (Jung et al., 2015; Kaitu’U-Lino et al., 2017) and Zfp36/TTP (Lai et al., 1999). Cyclooxigenase 2 was also upregulated (approx. 20-fold by IL-1), but only 2-fold more by PS, and therefore does not show up in Figure 2B as the threshold was set to 3-fold regulation by PS. NR4As have been shown to cross-talk with NF-κB and thereby modulate its activity (Murphy and Crean, 2015). For example, NR4A1 (Nurr77) suppressed EC activation though induction of IκBα expression (You et al., 2009). In astrocytes, NF-κB dependent inflammatory activation was inhibited by compensatory expression of NR4A1 and -2, indicating that they constitute a negative feedback loop (Popichak et al., 2018). NR4A3 over-expression inhibited the NF-κB signaling in a mouse model of myocardial infarction, by decreasing IκBα phosphorylation and inhibiting p65 nuclear translocation (Jiang et al., 2019). Lentiviral overexpression of all three factors reduced the expression of proinflammatory cytokines as well as the oxidized low-density lipoprotein uptake in human macrophages (Bonta et al., 2006). ATF3 has been shown to cross-talk to NF-κB and to act as a negative regulator of pro-inflammatory responses in different settings, such as preeclampsia (Kaitu’U-Lino et al., 2017), inflammation after cerebral injury (Wang et al., 2012), and Toll-like receptor 4 signaling (Gilchrist et al., 2006), in the latter case by direct binding to the p65/RelA subunit of NF-κB (Kwon et al., 2015). Zfp36/TTP, which on one hand binds to AUUUA rich elements in certain mRNAs leading to their destabilization (Lai et al., 1999), can also directly inhibit NF-κB (Schichl et al., 2009). GM-CSF (CSF2) is an example of a mRNA which is destabilized by TTP, suggesting that this could be a mechanism for the down-regulation of this growth factor (Carballo et al., 2000).

Taken together, transcription factors of the NR4A family and ATF3, as well as Zfp36/TTP have been associated at least in some cases with negative regulatory functions either in EC or other cell types, and also to cross-talk with NF-κB. Thus, a number of genes affected by PS have the potential or documented ability to inhibit NF-κB or other functions in EC. However, in general the PS sensitive genes are quite divergent in regard to their function, and this would predict that PS may preferentially inhibit certain aspects of the inflammatory episode, but could also promote others.

For a deeper insight into the mechanism(s) of PS-mediated inhibition we undertook a bioinformatics-based attempt and compared the promoter regions of PS sensitive and non-sensitive genes. This was based on the hypothesis that first, many genes are regulated on the transcriptional level, and second, specific transcription factor(s) as defined by the presence of their binding site(s) would be operative in the PS-sensitive gene set (or *vice versa*, ones with repressive function in the non-responsive set). In addition, target gene-micro RNA interactions were disclosed. As listed in Table 1, quantitative differences in NF-κB family member binding sites were identified. Taken together, we assume that NF-κB alone is not responsible for the early stages of inhibition, since activation of this pathway became apparent only after the first wave of activation, a time when differences in gene expression were already detectable. Alternatively, since gene expression is usually regulated through the interplay of different transcription factors binding to their promoters, the inhibition of NF-κB could in certain genes become compensated through the enhanced activity of other transcription factors. IL-8 could be an example of this scenario, as it contains NF-κB, AP-1 (a target of JNK) and NF-IL6 binding sites; here, the loss of NF-κB activity might be compensated by enhanced JNK signaling, however, this remains speculative until experimentally proven. The next most significant hits were STAT5a and the target(s) of MIR23 A, B. Whereas the former supports, together with the regulation of IRF1, a role of PS in interferon signaling, the latter has been reported to suppress Apaf-1. Interestingly, Apaf-1 is co-regulated together with the NF-κB inhibitor IKIP (Hofer-Warbinek et al., 2004), so it might be speculated that MIR23 A, B could as well regulate indirectly NF-κB. In the control pathway, besides other microRNAs, two transcriptional regulators/signaling pathways, namely CHOP and CREBP1/ATF2 are overrepresented; these findings reveal a complex interplay of different mechanism(s) warranting future studies on the level of the manifold PS constituents.

## Data Availability

The original contributions presented in the study are publicly available. This data can be found here: https://www.ncbi.nlm.nih.gov/geo/, GSE178106.
