# Supplementary material for: Pterocarpus santalinus Selectively Inhibits a Subset of Pro-Inflammatory Genes in Interleukin-1 Stimulated Endothelial Cells
Source: Front Pharmacol. 2022 Jan 18;12:802153. doi: 10.3389/fphar.2021.802153 (PMC8804362; doi:10.3389/fphar.2021.802153)
Supplement: Supplementary file 1 [file DataSheet1.docx]

*Supplementary Material*

**
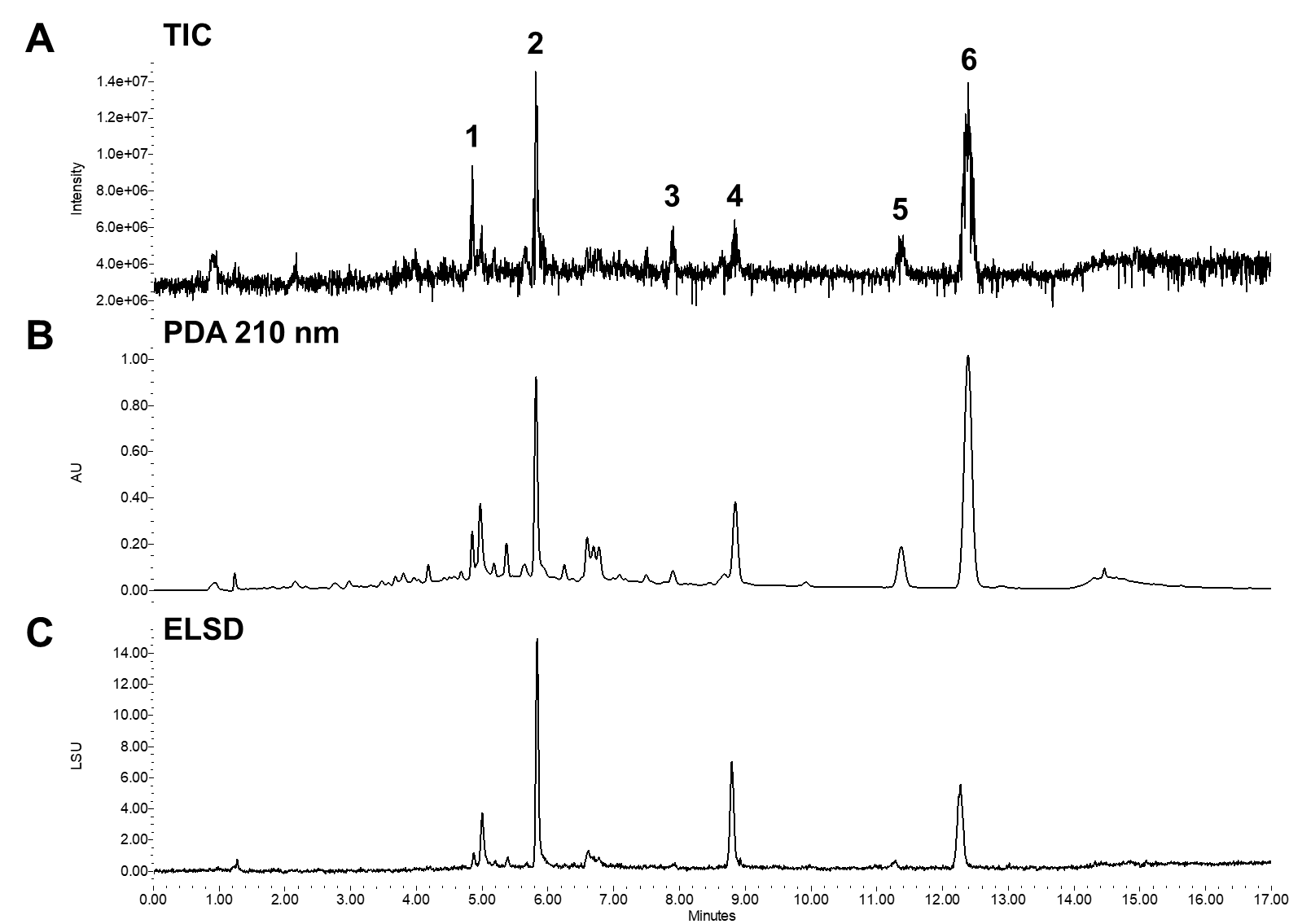
**

**Supplementary Figure S1.** UPLC chromatogram of PS showing the (**A**) total ion chromatogram (TIC, positive mode) with annotated compounds **1** – **6**, (**B**) PDA 210 nm, and (**C**) ELSD chromatogram. For the analytical characterization of PS a Waters Acquity UPLC H-Class was used. PS was chromatographed over a HSS T3 100 mm column using a binary mobile phase system consisting of A) H_2_O and B) CH_3_CN. The gradient was from 30%-98% B in 17 min followed by 5 min re-equilibration. Method in detail: 30% B isocratic for 0.5 min, 30%-45% B in 1.5 min, 45%-50% B in 1.5 min, 50%-55% B in 8.5 min, 55%-98% B in 0.1 min, 98% B isocratic for 3.9 min, 98%-30% B in 0.1 min, 30% isocratic for 0.9 min; Conditions: column temperature, 40 °C; flow rate, 0.250 mL/min; injection volume, 1 µL. Detection of compounds using photodiode array detector (PDA) and evaporative light scattering detector (ELSD). PDA conditions: 210 nm and full range spectra 192-400 nm. The UPLC system was further coupled to an Acquity QDa mass detector with an electrospray ionization source and an isocratic solvent manager as a make-up pump. A dereplication of PS in the positive ionization mode (*m*/*z* 100 – 1250) was performed using a make-up flow rate of 0.150 mL/min with 10 mM ammonium formate in a mixture of 95% H_2_O and 5% MeOH.

**Supplementary Table S1** Results from the dereplication of PS via literature search

|  | **Retention time**  **[min]** | ***m/z* value**  **[positive mode]** | **Estimated molecular weigth [g/mol]** | **Proposed compound** | **CAS Registry Number** |
| --- | --- | --- | --- | --- | --- |
| **1** | 4.837 | 331.2 | 330.29 | Santalin AC | 167425-76-5 |
| **2** | 5.800 | 301.2 | 300.26 | Santal | 529-60-2 |
| **3** | 7.861 | 315.2 | 314.29 | Melannein | 10386-55-7 |
| **4** | 8.794 | 257.3 | 256.30 | Pterostilbene | 537-42-8 |
| **5** | 11.270 | 299.3 | 298.29 | (-)-Pterocarpin | 524-97-0 |
| **6** | 12.266 | 285.3 | 284.31 | (-)-Homopterocarpin | 606-91-7 |


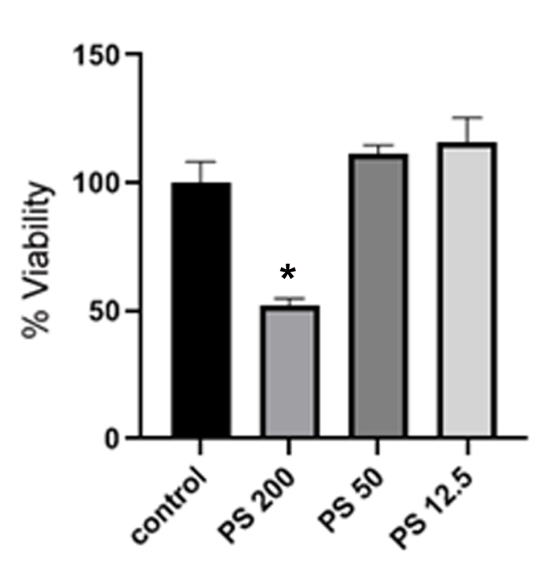


**Supplementary Figure S2.** Toxicity assay: HUVEC were incubated for 6 h with different concentrations of PS (i.e., 200, 50 and 12.5 µg/mL) or left untreated. Viability was assayed using the Resazorin assay. Bars represent mean ± SD. * indicate *p* < 0.05.

**Supplementary Table S2.** Primers for qPCR.

| **name** | **forward (5´-3´)** | **reverse (5´-3´)** |
| --- | --- | --- |
| **Glyceraldehyde**  **3-phosphate Dehydrogenase (GAPDH)** | AGAAGGCTGGGGCTCATTT | CTAAGCAGTTGGTGGTGCAG |
| **E-selectin (SELE)** | CCTGTGAAGCTCCCACTGA | GGCTTTTGGTAGCTTCCATCT |
| **Vascular Cell Adhesion Molecule 1 (VCAM1)** | CCGGCTGGAGATATTAC | TGTATCTCTGGGGGCAACAT |
| **TNF Receptor Associated Factor 1 (TRAF1** | CAGGAAGCCGTCTTCGAACT | TGAACCCCAACAGCAGGTTT |
| **C-X3-C Motif Chemokine Ligand 1**  **(CX3CL1, fractalkine)** | CACCACGGTGTGACGAAATG | ATCTGCTTCTCGAAGGTGCC |
| **Interferon Regulatory Factor 1 (IRF1)** | GCCCTCCACCTCTGAAGCTA | TACCCCTTCCCATCCACGTT |
| **Plasminogen Activator, Urokinase (PLAU)** | AGATGAAGCTTGCTTGGGTCA | GGACAGTGGCAGAGTTCCAG |
| **Tristetraprolin**  **(TTP, Zfp36)** | CATGGCCAACCGTTACACCA | CTCCATGGTCGGATGGCAC |
| **Nuclear Receptor Subfamily 4 Group A Member 1 (NR4A1)** | ATACACCCGTGACCTCAACC | CCCCTTCAGGCTGTCTGTTC |
| **Nuclear Receptor Subfamily 4 Group A Member 2 (NR4A2)** | ACAGACGCGGAGAACTCCTA | TGAGGCGAGGACCCATACTG |
| **Nuclear Receptor Subfamily 4 Group A Member 3 (NR4A3)** | AGGGCTGCAAGGGCTTTTT | ACGACCTCTCCTCCCTTTCA |
| **Basic Helix-Loop-Helix Family Member E40 (BHLHE40)** | TGCCCTGCAGAGTGGTTTAC | CTCGTGCTTGGCCAGATACT |
| **Interleukin-8**  **(IL-8, CXCL8)** | GTGTGAAGGTGCAGTTTTGC | AGTTTTCCTTGGGGTCCAGA |
| **A20, inhibitor of apoptosis** | CATGCATGCCACTTCTCAGT | CATGGGTGTGTCTGTGGAAG |

**Supplementary Table S3.** Official Gene Symbols and corresponding names of genes shown in Figure 4, and sets used for bioinformatic analysis by NetworkAnalyst.

| **Gene Symbol** | **Description** |
| --- | --- |
|  |  |
| **Figure 5A genes and induced set for Network Analyst** | |
| **TRAF1** | TNF receptor-associated factor 1 |
| **CX3CL1** | chemokine (C-X3-C motif) ligand 1 |
| **NUAK2** | NUAK family, SNF1-like kinase, 2 |
| **VCAM1** | vascular cell adhesion molecule 1 |
| **CSF2** | colony stimulating factor 2 (granulocyte-macrophage) |
| **BIRC3** | baculoviral IAP repeat containing 3 |
| **TNFRSF9** | tumor necrosis factor receptor superfamily, member 9 |
| **PLAU** | plasminogen activator, urokinase |
| **SERPINB2** | serpin peptidase inhibitor, clade B (ovalbumin), member 2 |
| **TNFAIP2** | tumor necrosis factor, alpha-induced protein 2 |
| **IL1B** | interleukin 1 beta |
| **CITED4** | Cbp/p300-interacting transactivator, with Glu/Asp rich carboxy-terminal domain, 4 |
| **IRF1** | interferon regulatory factor 1 |
| **CSF3** | colony stimulating factor 3 |
| **IRAK2** | interleukin 1 receptor associated kinase 2 |
| **TMEM217** | transmembrane protein 217 |
| **DUSP5** | dual specificity phosphatase 5 |
| **BIRC3** | Memczak2013 ANTISENSE, CDS, coding, INTERNAL best transcript NM_182962 |
| **SPRY4** | sprouty RTK signaling antagonist 4 |
| **S100A3** | S100 calcium binding protein A3 |
| **KITLG** | KIT ligand |
| **FOXC2** | forkhead box C2 |
| **PPP1R15B** | protein phosphatase 1, regulatory subunit 15B |
| **BMP2** | bone morphogenetic protein 2 |
| **ICAM1** | intercellular adhesion molecule 1 |
| **RNF19B** | ring finger protein 19B |
| **PPAP2B** | Transcript Identified by AceView, Entrez Gene ID(s) 8613 |
| **LRIG1** | Leucine Rich Repeats And Immunoglobulin Like Domains 1 |
| **SNX5** | Jeck2013 ALT_DONOR, coding, INTERNAL, intronic best transcript NM_152227 |
| **NFKBID** | nuclear factor of kappa light polypeptide gene enhancer in B-cells inhibitor, delta |
| **NOCT** | nocturnin |
| **RIPK2** | receptor-interacting serine-threonine kinase 2 |
| **KDM6B** | lysine (K)-specific demethylase 6B |
| **FJX1** | four jointed box 1 |
| **PTX3** | pentraxin 3, long |
| **BCL3** | B-cell CLL/lymphoma 3; microRNA 8085 |
| **USP12** | Transcript Identified by AceView, Entrez Gene ID(s) 219333 |
| **LGALSL** | lectin, galactoside-binding-like |
| **HIVEP2** | NM_006734 |
| **SAMD4A** | NM_015589 |
| **REL** | v-rel avian reticuloendotheliosis viral oncogene homolog |
| **ZBTB10** | zinc finger and BTB domain containing 10 |
| **EXT1** | NM_000127 |
| **EHD1** | EH domain containing 1 |
| **SLC7A2** | solute carrier family 7 (cationic amino acid transporter, y+ system), member 2 |
| **TNFAIP6** | tumor necrosis factor, alpha-induced protein 6 |
| **KLF7** | Kruppel-like factor 7 (ubiquitous) |
| **NFKB1** | nuclear factor of kappa light polypeptide gene enhancer in B-cells 1 |
| **SOX7** | SRY box 7 |
| **SAV1** | salvador family WW domain containing protein 1 |
| **CLDN1** | claudin 1 |
| **MED21** | mediator complex subunit 21 |
| **HBEGF** | heparin-binding EGF-like growth factor |
| **ZC3H12C** | zinc finger CCCH-type containing 12C |
| **IL1A** | interleukin 1 alpha |
|  |  |
| **Figure 5B genes** | |
| **ATF3** | activating transcription factor 3 |
| **DDIT4** | DNA damage inducible transcript 4 |
| **EGR1** | early growth response 1 |
| **ARRDC3** | arrestin domain containing 3 |
| **BHLHE40** | basic helix-loop-helix family, member e40 |
| **ZFP36** | ZFP36 ring finger protein |
| **NR4A1** | nuclear receptor subfamily 4, group A, member 1 |
| **NR4A2** | nuclear receptor subfamily 4, group A, member 2 |
|  |  |
| **Figure 5C genes** | |
| **HOXA11** | homeobox A11 |
| **SPRY1** | sprouty RTK signaling antagonist 1 |
| **C5orf66** | chromosome 5 open reading frame 66 |
| **CXCL13** | chemokine (C-X-C motif) ligand 13 |
| **MTCP1** | C-x(9)-C motif containing 4; mature T-cell proliferation 1 |
| **AMPD1** | adenosine monophosphate deaminase 1 |
| **PABPC4L** | poly(A) binding protein, cytoplasmic 4-like |
| **ZNF57** | zinc finger protein 57 |
| **IZUMO1** | izumo sperm-egg fusion 1 |
| **TTC31** | Memczak2013 ALT_ACCEPTOR, ALT_DONOR, coding, INTERNAL, intronic best transcript NM_022492 |
| **CLASP2** | Transcript Identified by AceView, Entrez Gene ID(s) 23122 |
| **TXNIP** | thioredoxin interacting protein |
| **ADM** | adrenomedullin |
| **ID1** | inhibitor of DNA binding 1, dominant negative helix-loop-helix protein |
| **C10orf10** | chromosome 10 open reading frame 10 |
| **ZNF792** | zinc finger protein 792 |
|  |  |
| **control gene set for NetworkAnalyst** | |
| **ITPKC** | inositol-trisphosphate 3-kinase C |
| **GK** | glycerol kinase |
| **VEGFA** | vascular endothelial growth factor A |
| **TGFB3** | transforming growth factor beta 3 |
| **CCL7** | chemokine (C-C motif) ligand 7 |
| **GDPD1** | glycerophosphodiester phosphodiesterase domain containing 1 |
| **CDKN1A** | cyclin-dependent kinase inhibitor 1A (p21, Cip1) |
| **IER3** | immediate early response 3 |
| **PTCD3** | pentatricopeptide repeat domain 3 |
| **CYR61** | cysteine-rich, angiogenic inducer, 61 |
| **TRAK2** | transcript NM_015049 |
| **TRIB1** | tribbles pseudokinase 1 |
| **RHOB** | ras homolog family member B |
| **GBP3** | NM_018284 |
| **CXCL8** | chemokine (C-X-C motif) ligand 8 |
| **AMTN** | amelotin |
| **KALRN** | Transcript Identified by AceView, Entrez Gene ID(s) 8997 |
| **FANK1** | fibronectin type III and ankyrin repeat domains 1 |
| **DNAL1** | dynein, axonemal, light chain 1 |
| **MAP3K8** | mitogen-activated protein kinase kinase kinase 8 |
| **CXCL3** | chemokine (C-X-C motif) ligand 3 |
| **PPP1R15A** | protein phosphatase 1, regulatory subunit 15A |
| **SIRT1** | sirtuin 1 |
| **ADAMTS1** | ADAM metallopeptidase with thrombospondin type 1 motif 1 |
| **NFKBIA** | nuclear factor of kappa light polypeptide gene enhancer in B-cells inhibitor, alpha |
| **CXCL2** | chemokine (C-X-C motif) ligand 2 |
| **RPGRIP1L** | RPGRIP1-like |
